# Diagnostic and prognostic value of circular RNA CDR1as/ciRS‐7 for solid tumours: A systematic review and meta‐analysis

**DOI:** 10.1111/jcmm.15619

**Published:** 2020-08-12

**Authors:** Yutian Zou, Shaoquan Zheng, Xinpei Deng, Anli Yang, Yanan Kong, Maryam Kohansal, Xiaoqian Hu, Xiaoming Xie

**Affiliations:** ^1^ Department of Breast Oncology Sun Yat‐sen University Cancer Center Guangzhou China; ^2^ State Key Laboratory of Oncology in South China Collaborative Innovation Center for Cancer Medicine Guangzhou China; ^3^ School of Medicine Sun Yat‐sen University Guangzhou China; ^4^ Department of Biology Payame Noor University Tehran Iran; ^5^ School of Biomedical Sciences The University of Hong Kong Hong Kong SAR China

**Keywords:** biomarker, cancer, CDR1as, circular RNA, ciRS‐7

## Abstract

The circular RNA, CDR1as/ciRS‐7, functions as a vital regulator in various cancers; however, the predictive value of CDR1as remains controversial. Therefore, a comprehensive analysis for clarifying the precise diagnostic and prognostic value of CDR1as in solid tumours is needed. A literature review of several databases was conducted for identifying potential studies. Pooled odds ratios (ORs) and hazard ratios (HRs) were used for evaluating the diagnostic accuracy variables and survival. Overall, 15 studies (1787 patients) and 11 studies (1578 patients) were included for diagnostic and prognostic outcome syntheses, respectively. Up‐regulated CDR1as expression was found to be correlated with worse clinicopathological characteristics, including the T status, N status, histological grade, TNM stage and distant metastasis. The synthesized sensitivity was 0.72 (95% confidence interval [CI], 0.65‐0.79), and the specificity was 0.80 (95% CI, 0.74‐0.86). The positive likelihood ratio (LR), negative LR and diagnostic odds ratio (DOR) were 3.70, 0.34 and 10.80, respectively. The area under the receiver operator characteristic curve was 0.84 (95% CI, 0.80‐0.87). In the pooled prognostic analysis, patients with high CDR1as expression had worse overall survival (HR = 2.40, *P* < 0.001) and disease‐free survival (HR = 1.74, *P* < 0.001). These results suggest that CDR1as is a reliable diagnostic and prognostic biomarker with high accuracy and efficiency, which may potentially facilitate clinical decisions on solid tumours in the future.

## 1 INTRODUCTION

Cancers are fatal diseases that are regarded as the leading cause of death according to the global statistics available from the World Health Organization.^1^ The latest data revealed that there are approximately 1 806 590 newly diagnosed cancer cases and 606 520 cancer deaths in the United States in 2020.^2^ Despite the considerable resources and expenditures devoted to therapeutic drug discovery and diagnostic biomarker development, the majority of cancers are still incurable, which remains a thorny problem in human health. Several biomarkers (alpha‐fetoprotein [AFP], carcinoembryonic antigen [CEA], etc) for tumour diagnosis have been developed and widely used in clinical practice; however, they are inefficient with low accuracy. Therefore, it is imperative to establish a diagnostic biomarker with higher accuracy in the early stage of cancer and develop more robust therapeutic methods.

Recently, as a novel endogenous non‐coding RNA, circular RNA (circRNA) has attracted the attention of many researchers and has rapidly become a heated topic in the field of biomedicine.^3^ Derived from the back‐splice of exons and/or introns of messenger RNAs, circRNAs are abundant and stable in mammalian tissues.^4^ With no 5′ cap and 3′‐poly‐A tail, circRNAs are once considered useless by‐products of incorrect splicing in cells.^5^ With the progress made in high‐throughput RNA sequencing and bioinformatic analysis, an increasing number of circRNAs have been captured and identified.^6^, ^7^ Scientists have discovered that circRNAs are versatile regulators of the process of multiple diseases, including diabetes mellitus, Alzheimer's disease, autism, heart failure and cancer.^8^, ^9^, ^10^, ^11^, ^12^ circRNAs serve as mediators of intracellular biological activities by different mechanisms, including binding proteins, sponging miRNAs and encoding short peptides.^13^ For example, circFBXW7 is down‐regulated in malignant tissues, which inhibits cell proliferation and invasion by encoding a 21kDa novel short peptide FBXW7‐185aa and sponging miR‐197‐3p in glioma and triple‐negative breast cancer.^14^, ^15^ A circRNA derived from CTNNB1 exons facilities cell proliferation and metastasis by encoding a novel 370‐amino acid CTNNB1 isoform and activating the Wnt signalling pathway in hepatocellular carcinoma.^16^ CircRAD18, circRNA FLI1 and circPLK1 were also identified as oncogenic drivers of breast cancer through different mechanisms, including reduction of apoptosis, maintenance of DNA methylation and activation of autophagy.^17^, ^18^, ^19^

Originating from CDR1AS (cerebellar degeneration‐related protein 1 antisense transcript), CDR1as is the most well‐known circRNAs that has been studied in many disease models. CDR1as contains >70 conventional binding sites of miR‐7 and serves as a negative regulator of miR‐7 for altering the expression of multiple key target genes.^20^ Because of its unique characteristic, CDR1as is also termed as ciRS‐7, the circRNA sponge for miR‐7. CDR1as has been widely studied and shown to be an oncogenic factor in various cancers. For example, CDR1as sponges miR‐7 to facilitate colorectal cancer growth and invasion by regulating EGFR signalling pathway activity.^21^ By inhibiting miR‐876‐5p, CDR1as promotes the growth and metastatic ability of oesophageal squamous cell carcinoma and up‐regulates the expression of the MAGE‐A family.^22^ Additionally, CDR1as has the potential to regulate the tumour environment, which is negatively correlated with immune cell infiltration and immune response.^23^ Thus, CDR1as has great potential as an accurate diagnostic biomarker and novel therapeutic target for multiple cancers.

In the current study, we conducted a systematic review and meta‐analysis of reported studies for evaluating the diagnostic and prognostic value of CDR1as in solid tumours. Several indexes were analysed to assess whether CDR1as can be used as an ideal biomarker for diagnostic and prognostic prediction in solid tumours.

## 2 METHODS

### 2.1 Search strategy

A comprehensive search of PubMed, Embase, Cochrane Library and Web of Science online databases was performed. The website addresses of each database are presented in Table S5. The period of literature retrieval was from 1 January 1990 to 30 September 2019. The following keywords were used in the retrieval strategy: “CDR1as” or “ciRS‐7” or “hsa_circ_0001946.” All search strategies were conducted following the Preferred Reporting Items for Systematic Reviews and Meta‐Analyses (PRISMA) guidelines.^24^

### 2.2 Inclusion and exclusion criteria

Studies concerning the prognostic or diagnostic value of CDR1as/ciRS‐7 expression in patients with cancer were eligible for quantitative synthesis. Only articles published in the English and Chinese languages were included in this study. The exclusion criteria were review, case reports, letters, and commentaries and studies with insufficient or ambiguous data or not relevant to ciRS‐7 study in cancer. An insufficient data study was defined as a study with neither diagnostic accuracy data nor prognostic outcomes of ciRS‐7. An ambiguous data study was defined as a study with wrong statistics. Studies of other biomarkers (lncRNAs, other circRNAs, etc) or non‐cancer diseases were also excluded in this research, which were defined as not relevant to ciRS‐7 study in cancer.

### 2.3 Study selection

All search results were independently examined by two authors (YTZ and XPD) with discrepancies consulted by a third reviewer (SQZ). The selection criteria were applied by reviewers after screening the potentially included studies. Duplicates were removed using Endnote X9 software or manually.

### 2.4 Data extraction

The baseline characteristics of each study (authors, year of publication, cancer type, sample size, specimen sources, detective method and follow‐up) were recorded independently by two reviewers. The correlation between CDR1as expression and clinicopathological characteristics, including age, sex, T status, N status, grade, distant metastasis and TNM stage, was also evaluated. Odds ratio (OR) and 95% confidence interval (CI) were used to describe the abovementioned information. To evaluate the diagnostic accuracy, the true positive (TP), false positive (FP), false negative (FN), true negative (TN) and area under the curve (AUC) in each study were recorded to synthesize the sensitivity, specificity, positive likelihood ratio (LR), negative LR, diagnostic odds ratio (DOR) and AUC. The information was also obtained by contacting the author or extracting data from the scatter plot if the number was unavailable from the text. Prognostic outcomes were overall survival (OS) and disease‐free survival (DFS) reported as hazard ratios (HRs) and 95% CI according to the multivariate analysis. Moreover, the HRs were extracted from the studies that only presented Kaplan‐Meier survival curves using the method provided by Guyot et al.^25^

### 2.5 Methodology quality assessment

Methodology quality was assessed using the Newcastle‐Ottawa Scale (NOS) for prognostic cohort studies and the Quality Assessment of Diagnostic Accuracy Studies 2 (QUADAS‐2) for diagnostic studies. As for NOS, studies that scored 0‐6 were regarded as low‐quality research, while those that scored 7‐9 were defined as high‐quality evidence. A study with at least three unclear or high risk of bias was considered to have low quality, assessed using QUADAS‐2.

### 2.6 Data synthesis and analysis

HRs and ORs extracted from studies were synthesized using the random‐effects model in Review Manager software (version 5.3). Estimation of heterogeneity was performed using Cochran's Q test, which reported a *P*‐value and *I^2^* statistic. A *P*‐value < 0.1 or *I^2^* statistic > 50% indicated heterogeneity. In addition, publication bias was evaluated by inspecting the funnel plots and using Begg's test. Pooled sensitivity, specificity, positive LR, negative LR, DOR and AUC were calculated using the Stata software (version 15.1). Deeks’ funnel plot test and bivariate boxplot were employed to assess publication bias. Subgroup analysis was conducted to determine the potential sources of heterogeneity. Fagan's nomogram was used to predict post‐test probabilities.

## 3 RESULTS

### 3.1 Baseline characteristics of included studies

**FIGURE 1:**
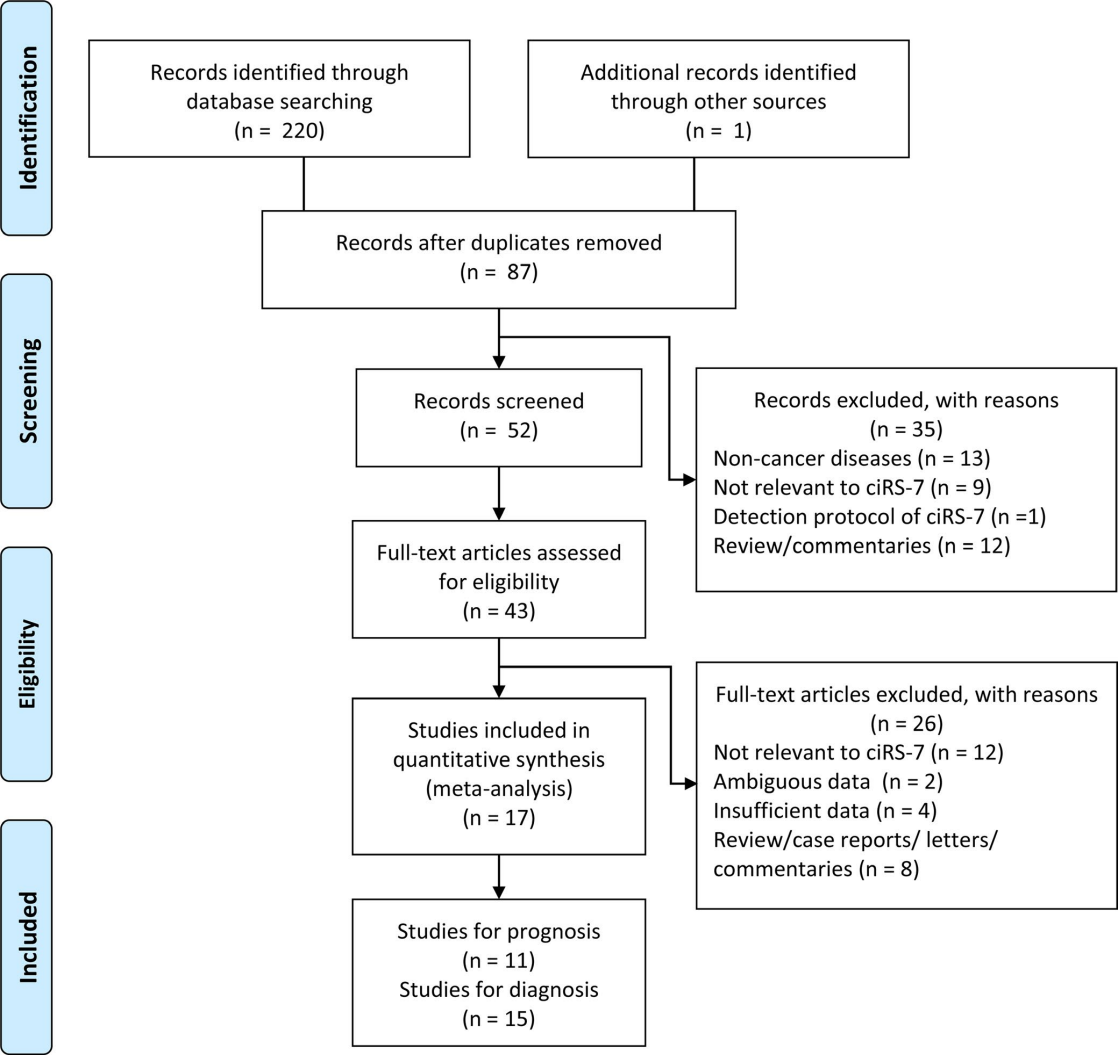
PRISMA flow diagram of the article retrieval strategy in this meta‐analysis

Our retrieval strategy identified 220 potentially relevant articles in total. The PRISMA flow diagram demonstrates the detailed process of study selection (Figure 1). Overall, 15 studies (1,787 patients) and 11 studies (1,578 patients) were included for diagnostic and prognostic outcome synthesis, respectively. Among these studies, nine different cancer types were involved in the result analysis, including oesophageal squamous cell carcinoma (ESCC) in four studies,^26^, ^27^ non–small cell lung cancer (NSCLC) in three studies,^28^, ^29^, ^30^ colorectal cancer (CC) in two studies,^21^, ^22^, ^23^, ^24^, ^25^, ^26^, ^27^, ^28^, ^29^, ^30^, ^31^ hepatocellular carcinoma (HCC) in two studies,^32^, ^33^ triple‐negative breast cancer (TNBC) in one study,^34^ osteosarcoma (OS) in one study,^35^ laryngeal squamous cell carcinoma (LSCC) in one study,^36^ cholangiocarcinoma (CHOL) in one study ^37^ and cervical squamous cell carcinoma (CESC) in one study.^38^ Additionally, real‐time polymerase chain reaction (qRT‐PCR) analysis was used to detect CDR1as expression. Detailed baseline information of the included studies is presented in Tables S1 and S2. The specific mechanisms and egulated pathways of CDR1as in each study are summarized in Table S3. Quality assessment results are shown in Table S4 using the Newcastle‐Ottawa Scale for cohort prognostic studies and in Figures S1 and S2 using QUADAS‐2 for diagnostic studies.

### 3.2 Clinicopathological parameters of CDR1as in cancers

In the pooled analyses of clinicopathological characteristics of CDR1as in solid tumours, a significant association between CDR1as expression and clinicopathological parameters was revealed in our results (Table 1). Tumours with higher CDR1as expression had more advanced T status (OR = 3.36; 95% CI, 1.58‐7.15; *P* = 0.002), N status (OR = 1.97; 95% CI, 1.46‐2.66; *P* < 0.001), histological grade (OR = 2.37; 95% CI, 1.56‐3.59; *P* < 0.001), and TNM stage (OR = 2.60; 95% CI, 1.93‐3.90; *P* < 0.001) and higher risk of distant metastasis (OR = 2.23; 95% CI, 1.24‐4.00; *P* = 0.007). Additionally, there were no significant associations between CDR1as expression and other clinicopathological parameters, including age (OR = 1.17; 95% CI, 0.84‐1.63; *P* = 0.34) and gender (OR = 1.11; 95% CI, 0.83‐1.49; *P* = 0.48) in solid tumours.

**TABLE 1:** Correlation between CDR1as expression and clinicopathological characteristics of patients with cancers

	High CDR1as group	Low CDR1as group	No. of studies	No. of patients	Odds ratio (95% CI) High vs. Low	*P*	*I* ^2^ (%)
Age (years)							
Young (<50/60)	36%	37%	7	683	1 [Reference]	0.34	0
Old (≥50/60)	64%	63%			1.17 [0.84,1.63]		
Gender							
Male	55%	59%	8	830	1 [Reference]	0.48	0
Female	45%	41%			1.11 [0.83,1.49]		
T status							
T1‐2	64%	90%	2	224	1 [Reference]	0.002*	0
T3‐4	36%	10%			3.36 [1.58, 7.15]		
N status							
N0	44%	59%	7	776	1 [Reference]	<0.001*	70
N1‐3	56%	41%			1.97 [1.46,2.66]		
Histological grade							
G1‐2	55%	74%	4	438	1 [Reference]	<0.001*	76
G3	45%	26%			2.37 [1.56, 3.59]		
Distant metastasis							
Negative	82%	91%	3	452	1 [Reference]	0.007*	0
Positive	18%	9%			2.23 [1.24, 4.00]		
TNM stage							
I‐II	41%	64%	7	770	1 [Reference]	<0.001*	0
III‐IV	59%	36%			2.60 [1.93,3.90]		

* *P* < 0.05, statistically significant.

### 3.3 Diagnostic significance of CDR1as in cancers

**FIGURE 2:**
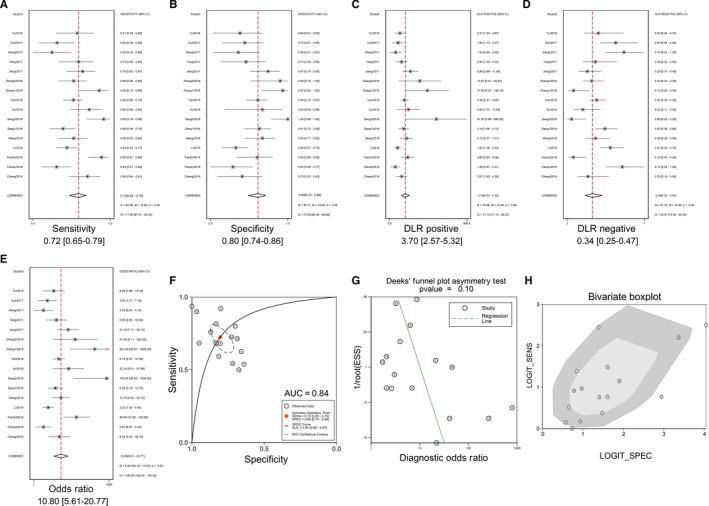
Forest plots evaluating the diagnostic value of CDR1as in cancers. A, Sensitivity; B, specificity; C, positive LR; D, negative LR; E, DOR; F, AUC; G, Deeks’ funnel plot; and H, bivariate boxplot. Abbreviations: AUC, area under curve; DOR, diagnostic odds ratio; LR, likelihood ratio

**FIGURE 3:**
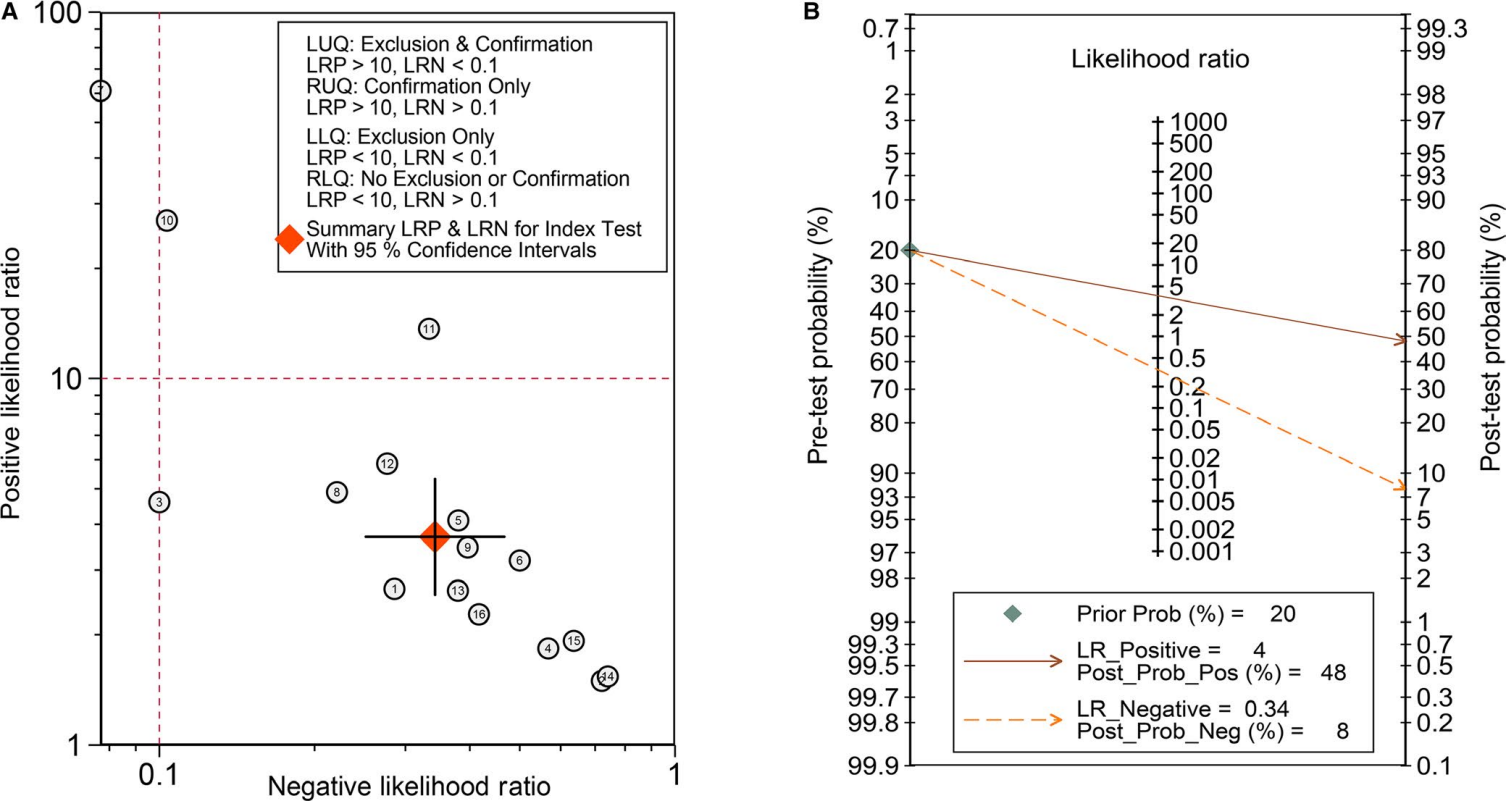
Likelihood ratio of CDR1as as an index of diagnosis. A, Scatter plot of positive and negative likelihood ratios with combined summary points. B, Fagan's nomogram was constructed to calculate the post‐test probabilities of CDR1as

The diagnostic accuracy of CDR1as was evaluated among 15 studies involving nine types of cancer. The synthesized sensitivity of all studies was 0.72 (95% CI, 0.65‐0.79), and the specificity was 0.80 (95% CI, 0.74‐0.86) (Figure 2A‐B). The positive LR, negative LR and DOR were 3.70 (95% CI, 2.57‐5.32), 0.34 (95% CI, 0.25‐0.47) and 10.80 (95% CI, 5.61‐20.77), respectively (Figure 2C‐E). The AUC of the summary receiver operator characteristic (sROC) curve was 0.84 (95% CI, 0.80‐0.87) (Figure 2F). Subgroup analyses showed an unsatisfactory diagnostic value in digestive system cancers (AUC = 0.72), and a significant decrease in heterogeneity was observed in each group (Table 2). Additionally, we found a higher diagnostic accuracy of CDR1as in plasma (AUC = 0.89) compared to tumour tissue (AUC = 0.66) in patients with ESCC despite a small sample size (Table 2). Deeks’ funnel plot asymmetry test revealed no publication bias (*P* = 0.10), and the bivariate boxplot manifested heterogeneous statistics (Figure 2G‐H). A scatter plot of positive and negative LRs with combined summary points is shown in Figure 3A. Fagan's nomogram was constructed to calculate post‐test probabilities of CDR1as, from which we found that the post‐test probability increased to 48% with a positive LR of 4, and the post‐test probability decreased to 8% with a negative LR of 0.34 (Figure 3B). These results indicate that CDR1as is a reliable diagnostic biomarker with high accuracy and efficiency.

**TABLE 2:** Summary of subgroup analysis for diagnostic accuracy of CDR1as in cancers

Study groups	No. of studies	Sensitivity (95% CI)	*I* ^2^ (%)	Specificity (95% CI)	*I* ^2^ (%)	Positive LR (95% CI)	*I* ^2^ (%)	Negative LR (95% CI)	*I* ^2^ (%)	DOR (95% CI)	*I* ^2^ (%)	AUC (95% CI)
All studies	15	0.72 (0.65‐0.79)	78	0.80 (0.74‐0.86)	74	3.70 (2.57‐5.32)	71	0.34 (0.25‐0.47)	83	10.80 (5.61‐20.77)	100	0.84 (0.80‐0.87)
Cancer type												
Digestive system	9	0.63 (0.58‐0.68)	44	0.74 (0.68‐0.80)	56	2.45 (1.86‐3.23)	32	0.50 (0.41‐0.60)	56	4.93 (3.12‐7.79)	90	0.72 (0.68‐0.76)
ESCC	4	0.61 (0.56‐0.67)	11	0.78 (0.64‐0.82)	74	2.37 (1.62‐3.47)	49	0.52 (0.42‐0.64)	54	4.54 (2.56‐8.08)	87	0.66 (0.61‐0.70)
NSCLC	2	0.68 (0.58‐0.78)	0	0.85 (0.77‐0.92)	66	3.81 (3.46‐4.17)	90	0.38 (0.28‐0.47)	0	10.95 (9.90‐11.39)	99	0.83 (0.78‐0.88)
CC	2	0.66 (0.49‐0.84)	24	0.71 (0.55‐0.86)	0	2.10 (1.71‐2.48)	67	0.64 (0.35‐0.93)	18	4.32 (3.65‐4.98)	88	0.68 (0.56‐0.81)
HCC	2	0.64 (0.47‐0.82)	0	0.71 (0.56‐0.86)	0	2.09 (1.72‐2.47)	0	0.57 (0.27‐0.88)	0	4.03 (3.37, 4.70)	0	0.71 (0.58‐0.83)
TNBC	1	0.94 (0.79‐0.99)	/	1.00 (0.89‐1.00)	/	61.00 (3.89‐956.55)	/	0.08 (0.02‐0.25)	/	793.00 (36.58‐1000.00)	/	0.98 (0.96‐1.00)
OS	1	0.82 (0.66‐0.92)	/	0.83 (0.59‐0.96)	/	4.89 (1.72‐13.90)	/	0.22 (0.11‐0.45)	/	22.14 (2.01‐97.89)	/	0.86 (0.76‐0.95)
LSCC	1	0.90 (0.73‐0.98)	/	0.97 (0.83‐1.00)	/	27.00 (3.92‐186.15)	/	0.10 (0.04‐0.30)	/	261.00 (25.57‐1000.00)	/	0.98 (0.95‐1.00)
CHOL	1	0.76 (0.62‐0.87)	/	0.87 (0.75‐0.95)	/	5.86 (2.89‐11.88)	/	0.28 (0.17‐0.45)	/	21.18 (7.71‐58.13)	/	0.86 (0.79‐0.94)
CESC	1	0.80 (0.64‐0.91)	/	0.70 (0.53‐0.83)	/	2.67 (1.62‐4.39)	/	0.29 (0.15‐0.55)	/	9.33 (3.34‐26.10)	/	0.80 (0.71‐0.89)
Sample size												
<100	9	0.75 (0.64‐0.84)	78	0.83 (0.71‐0.91)	76	4.57 (2.23‐9.38)	69	0.29 (0.18‐0.49)	84	15.49 (4.75‐50.57)	100	0.86 (0.83‐0.89)
≥100	7	0.65 (0.59‐0.70)	43	0.78 (0.70‐0.84)	74	2.89 (1.99‐4.21)	63	0.45 (0.36‐0.57)	69	6.40 (3.57‐11.50)	99	0.74 (0.70‐0.78)
Study quality												
High	8	0.72 (0.65‐0.78)	66	0.79 (0.74‐0.84)	64	3.42 (2.62‐4.47)	48	0.36 (0.28‐0.46)	68	9.55 (6.01‐15.20)	100	0.82 (0.79‐0.86)
Low	8	0.74 (0.57‐0.86)	87	0.82 (0.67‐0.91)	82	4.16 (1.81‐9.54)	82	0.31 (0.16‐0.63)	69	13.22 (2.97‐58.92)	100	0.85 (0.82‐0.88)
Publication year												
Before/in 2017	5	0.65 (0.55‐0.74)	62	0.74 (0.66‐0.81)	37	2.55 (1.73‐3.75)	19	0.47 (0.34‐0.66)	70	5.44 (2.70‐10.97)	99	0.76 (0.72‐0.80)
After/in 2018	11	0.76 (0.66‐0.83)	84	0.83 (0.75‐0.90)	82	4.57 (2.70‐7.73)	81	0.29 (0.19‐0.44)	88	15.76 (6.36‐39.09)	100	0.87 (0.83‐0.89)
Sample (ESCC)												
Tissue	4	0.61 (0.56‐0.67)	11	0.78 (0.64‐0.82)	74	2.37 (1.62‐3.47)	49	0.52 (0.42‐064)	54	4.54 (2.56‐8.08)	87	0.66 (0.61‐0.70)
Plasma	1	0.92 (0.81‐0.98)	/	0.80 (0.66‐0.90)	/	4.60 (2.63‐8.06)	/	0.10 (0.04‐0.26)	/	46.00 (13.38‐158.09)	/	0.89 (NA)

Abbreviations: AUC, area under curve; CC, colorectal cancer; CESC, cervical squamous cell carcinoma; CHOL, cholangiocarcinoma; ESCC, oesophageal squamous cell carcinoma; HCC, hepatocellular carcinoma; LSCC, laryngeal squamous cell carcinoma; NSCLC, non–small cell lung cancer; OS, osteosarcoma; TNBC, triple‐negative breast cancer.

### 3.4 Prognostic significance of CDR1as in cancers

**FIGURE 4:**
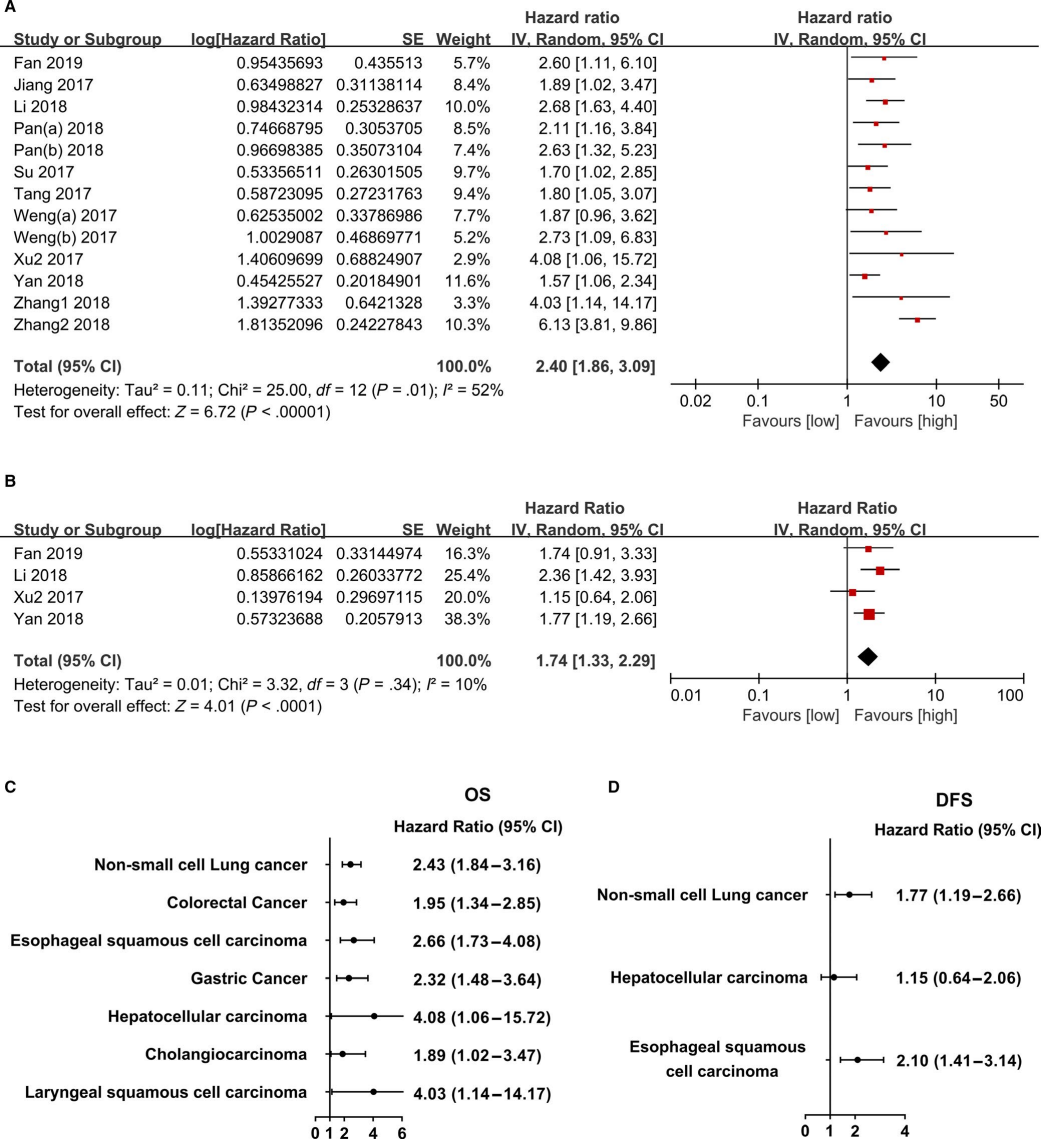
Forest plot evaluating the association between CDR1as expression and prognostic parameters in cancers. A, OS for all studies; B, DFS for all studies; C, OS for certain cancers and D, DFS for certain cancers. Abbreviations: DFS, disease‐free survival; OS, overall survival

Pooled analyses of 11 studies involving 1,578 patients indicated worse OS in tumours with high CDR1as expression (HR = 2.40; 95% CI, 1.86‐3.09; *P* < 0.001; *I^2^* = 52%) (Figure 4A). Consistently, CDR1as overexpression was significantly correlated with poorer DFS outcome in cancers (HR = 1.74; 95% CI, 1.33‐2.29; *P* < 0.001; *I^2^* = 10%) (Figure 4B). We further performed subgroup analyses to address the source of heterogeneity and determine its expression in specific subgroups. CDR1as expression was shown to be a risk factor for OS in all cancers included in this study and a prognostic factor for DFS in certain types of cancer (Figure 4C‐D). Moreover, subgroup analyses of the two groups based on sample size, univariate/multivariate analysis, follow‐up duration, quality of evidence and publication year revealed a decrease in heterogeneity in each group (Table 3). Funnel plots and Begg's test (*P* = 0.651) manifested no potential publication bias in the pooled analysis (Figure S3).

**TABLE 3:** Summary of subgroup analysis for OS comparing high CDR1as and low CDR1as expression group in patients with various cancers

	No. of studies	No. of patients	Hazard ratios (95% CI)	*P*	*I* ^2^ (%)
Overall	11	1428	2.34 (1.98, 2.77)	<0.001	52
NSCLC	3	320	2.43 (1.87, 3.16)	<0.001	90
CC	2	500	1.95 (1.34, 2.85)	<0.001	0
ESCC	2	173	2.66 (1.73, 4.08)	<0.001	0
GC	2	256	2.32 (1.48, 3.64)	<0.001	0
HCC	1	95	4.08 (1.06, 15.72)	0.04	/
CHOL	1	54	1.89 (1.02, 3.47)	0.04	/
LSCC	1	30	4.03 (1.14, 14.17)	0.03	/
Digestive system cancer	7	1078	2.24 (1.80, 2.80)	<0.001	0
Studies with > 100 patients	6	1139	1.97 (1.62, 2.40)	<0.001	0
Studies with ≤ 100 patients	5	289	3.72 (2.70, 5.13)	<0.001	59
Univariate analysis	10	1398	2.41 (2.05, 2.84)	<0.001	0
Multivariate analysis	9	990	2.43 (2.00, 2.94)	<0.001	62
Follow‐up > 60 mo	5	728	2.64 (2.10, 3.32)	<0.001	75
Follow‐up ≤ 60 mo	6	700	2.05 (1.61, 2.61)	<0.001	0
High‐quality study	4	668	2.04 (1.57, 2.65)	<0.001	6
Low‐quality study	7	760	2.34 (1.98, 2.77)	<0.001	63
Published in/before 2017	5	777	1.93 (1.47, 2.52)	<0.001	0
Published in/after 2018	6	651	2.65 (2.14, 3.29)	<0.001	69

Abbreviations: CC, colorectal cancer; CHOL, cholangiocarcinoma; ESCC, oesophageal squamous cell carcinoma; GC, gastric cancer; HCC, hepatocellular carcinoma; LSCC, laryngeal squamous cell carcinoma; NSCLC, non–small cell lung cancer; OS, overall survival.

## 4 DISCUSSION

In this study, we conducted a systematic review and meta‐analysis of reported studies to assess the diagnostic and prognostic values of CDR1as for solid tumours. Several outcomes were analysed to evaluate whether there is sufficient sensitivity and specificity for the diagnosis of solid tumours. Our retrieval strategy identified a total of 11 studies and 15 studies for prognostic and diagnostic outcome synthesis, respectively. The abnormal expression of CDR1as was observed in tumour tissues and even plasma samples from patients. First, we performed a pooled analysis to assess the correlation between CDR1as expression level and clinicopathological parameters. In summary, tumours with higher CDR1as expression had more advanced T status, N status, histological grade, and TNM stage and higher risk of distant metastasis. CDR1as is up‐regulated, which has been confirmed to exert influences on cell proliferation, migration, invasion and apoptosis in various tumours. The strong correlation between CDR1as expression and clinicopathological factors is possibly because of the versatile biological functions of CDR1as in different cancer cells. In the pooled analysis of diagnostic significance, CDR1as showed high sensitivity of 0.72 (95% CI, 0.65‐0.79) and specificity of 0.80 (95% CI, 0.74‐0.86) in distinguishing tumour tissues from adjacent normal tissues. In the analysis of specific cancers, CDR1as had the highest sensitivity and specificity in the diagnosis of triple‐negative breast cancer. Additionally, CDR1as had a specificity of 74% in diagnosing digestive system‐derived tumours. Therefore, detection of CDR1as can compensate for the lack of accuracy in digestive system neoplasm screening. The AUC of the ROC curve represents the comprehensive accuracy rate of detection. According to the pooled analysis, detection of CDR1as had an AUC of 0.84 (95% CI, 0.80‐0.87) in all included studies. The high AUC value of CDR1as indicates its high ability to distinguish benign from malignant tumours. To obtain appropriate sensitivity and specificity, further studies focusing on the selection of threshold values are expected in the future. In the subgroup analysis, CDR1as did not perform well in the diagnostic test of ESCC with an AUC of 0.66. However, a higher diagnostic accuracy of CDR1as was found in plasma (AUC = 0.89) compared to that in tumour tissue (AUC = 0.66) in patients with ESCC. Although it is an interesting finding, this outcome needs further verification owing to the small sample size. Based on the current knowledge, circRNAs have been shown to be present in exosomes, which can be secreted into plasma and urine.^39^, ^40^, ^41^ Owing to the advantages in the detection of early disease, liquid biopsy is currently a controversial topic in the field of malignancy diagnosis and surveillance. Detection of CDR1as in serum or urine provides a new strategy for the development of liquid biopsy biomarkers for cancers in the future. Additionally, we found that high CDR1as expression was associated with worse OS (HR = 2.40; 95% CI, 1.86‐3.09; *P* < 0.001) and DFS (HR = 1.74; 95% CI, 1.33‐2.29; *P* < 0.001) in synthesized analysis with low heterogeneity. However, CDR1as was not an independent prognostic factor for DFS in patients with hepatocellular carcinoma in the subgroup analysis, possibly because of the small sample size in the cohorts. Generally, our study showed that CDR1as is a great diagnostic and predictive biomarker for solid tumours with high accuracy and efficiency.

According to the definition of the National Cancer Institute, biomarkers are biological molecules found in blood, other body fluids or tissues, which is a sign of a normal or abnormal process or a condition or disease. A qualified biomarker possesses features including stability, sensitivity, specificity, accuracy and reproducibility.^42^ Several biomarkers for tumour diagnosis and surveillance have been widely used in clinical practice; however, their sensitivities and specificities are low. CEA, a recommended biomarker for the detection of colorectal cancer recurrence, has a pooled sensitivity of 71% and specificity of 88% when applying a threshold of 5 µg/L, as reported in a meta‐analysis of 23 studies.^43^ Another tumour marker, cancer antigen 125, is not able to reduce the mortality rate in screening for ovarian cancer according to four high‐quality clinical trials.^44^ Combined with ultrasound measurement in detecting early stage hepatocellular carcinoma, AFP is still inefficient with extremely low sensitivity (63%), which leads to a high misdiagnosis rate.^45^ As newly discovered non‐coding RNAs, an increasing number of circRNAs have been identified as promising biomarkers for cancer.^46^, ^47^ Compared to other biomarkers (protein, lncRNAs), circRNAs are more ubiquitous and stable with a closed‐loop structure in mammalian tissues.^5^ The predictive value of CDR1as as a biomarker for solid tumours was preliminarily explored in this study. Compared to some existing biomarkers, CDR1as is universally and specifically expressed in cancer tissues, which lays a solid foundation for its future development as a biomarker.

Several limitations exist in this study. First, because of the lack of prospective and double‐blind studies in diagnostic value investigation, bias was inevitable to some extent. Second, the small number of samples in each study contributed to the high heterogeneity in the analysis of diagnostic variables. Third, most samples are validated in Chinese cohorts, which results in population bias. Therefore, more multicentre and prospective diagnostic studies with high quality, large sample size and strict operation are required for further verification.

In conclusion, our study indicates that circRNA CDR1as has a remarkable association between its abnormal expression and clinicopathological, diagnostic and prognostic roles in patients with solid tumours. CDR1as is a promising diagnostic and predictive biomarker with high accuracy and efficiency which has potential to be an ideal indicator for solid tumours in the future.

## CONFLICT OF INTEREST

The authors declare no potential competing interests.

## AUTHOR CONTRIBUTION

**Yutian Zou:** Data curation (equal); Methodology (equal); Software (equal); Visualization (equal); Writing‐original draft (lead). **Shaoquan Zheng:** Data curation (equal); Formal analysis (equal); Investigation (equal); Software (equal). **Xinpei Deng:** Data curation (equal); Resources (equal); Software (equal); Validation (equal); Visualization (equal). **Anli Yang:** Formal analysis (equal); Resources (equal); Validation (equal). **Yanan Kong:** Formal analysis (equal); Validation (equal). **Maryam Kohansal:** Writing‐review & editing (equal). **Xiaoqian Hu:** Conceptualization (equal); Methodology (equal); Project administration (equal); Supervision (equal); Writing‐review & editing (equal). **Xiaoming Xie:** Conceptualization (lead); Funding acquisition (lead); Project administration (lead); Supervision (equal); Writing‐review & editing (equal). 

## ETHICAL APPROVAL

This article does not contain any studies with human participants performed by any of the authors.

## Supporting information

Fig S1

Fig S2

Fig S3

Table S1‐S5
