# Supplementary figures and images for: Diagnostic and prognostic value of circular RNA CDR1as/ciRS‐7 for solid tumours: A systematic review and meta‐analysis
Source: J Cell Mol Med. 2020 Aug 12;24(17):9507–17. doi: 10.1111/jcmm.15619 (PMC7520288; doi:10.1111/jcmm.15619)

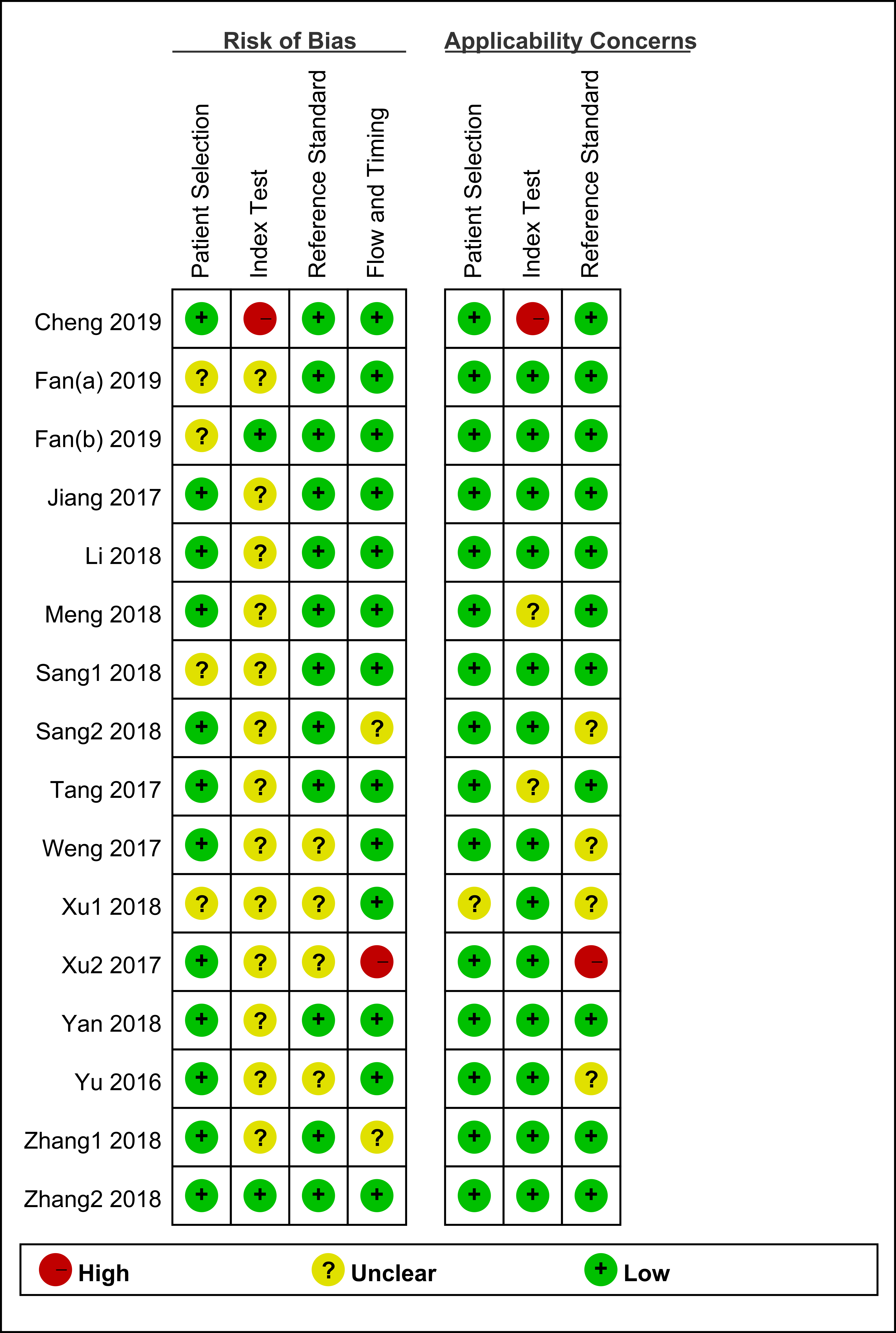

Supplement: Supplementary file 1 — Fig S1 [file JCMM-24-9507-s001.tif]

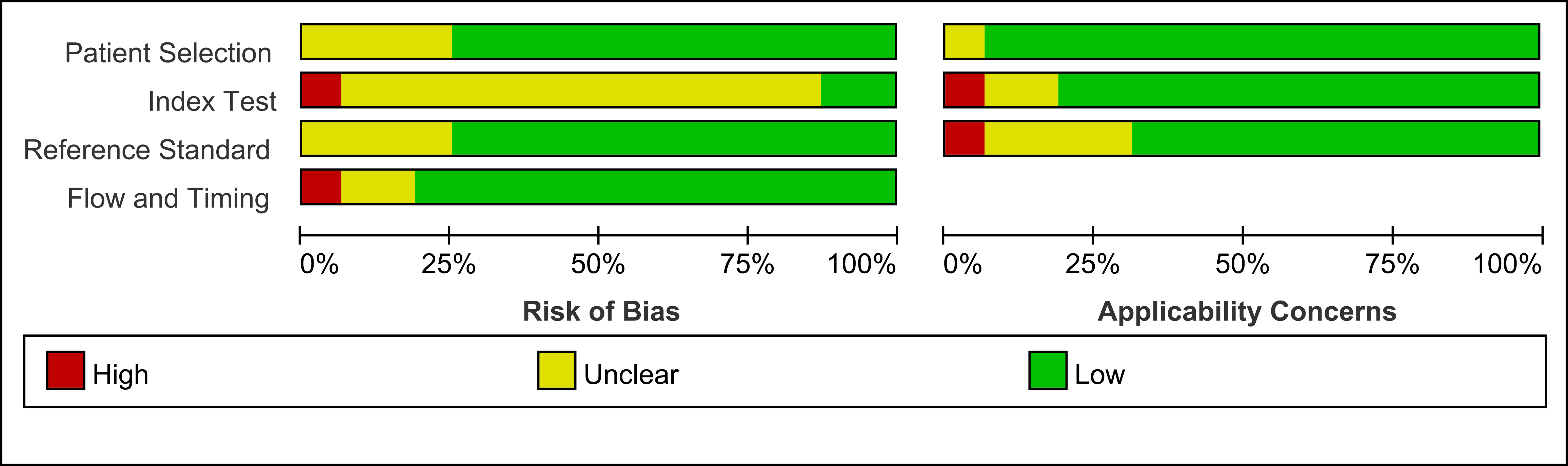

Supplement: Supplementary file 2 — Fig S2 [file JCMM-24-9507-s002.tif]

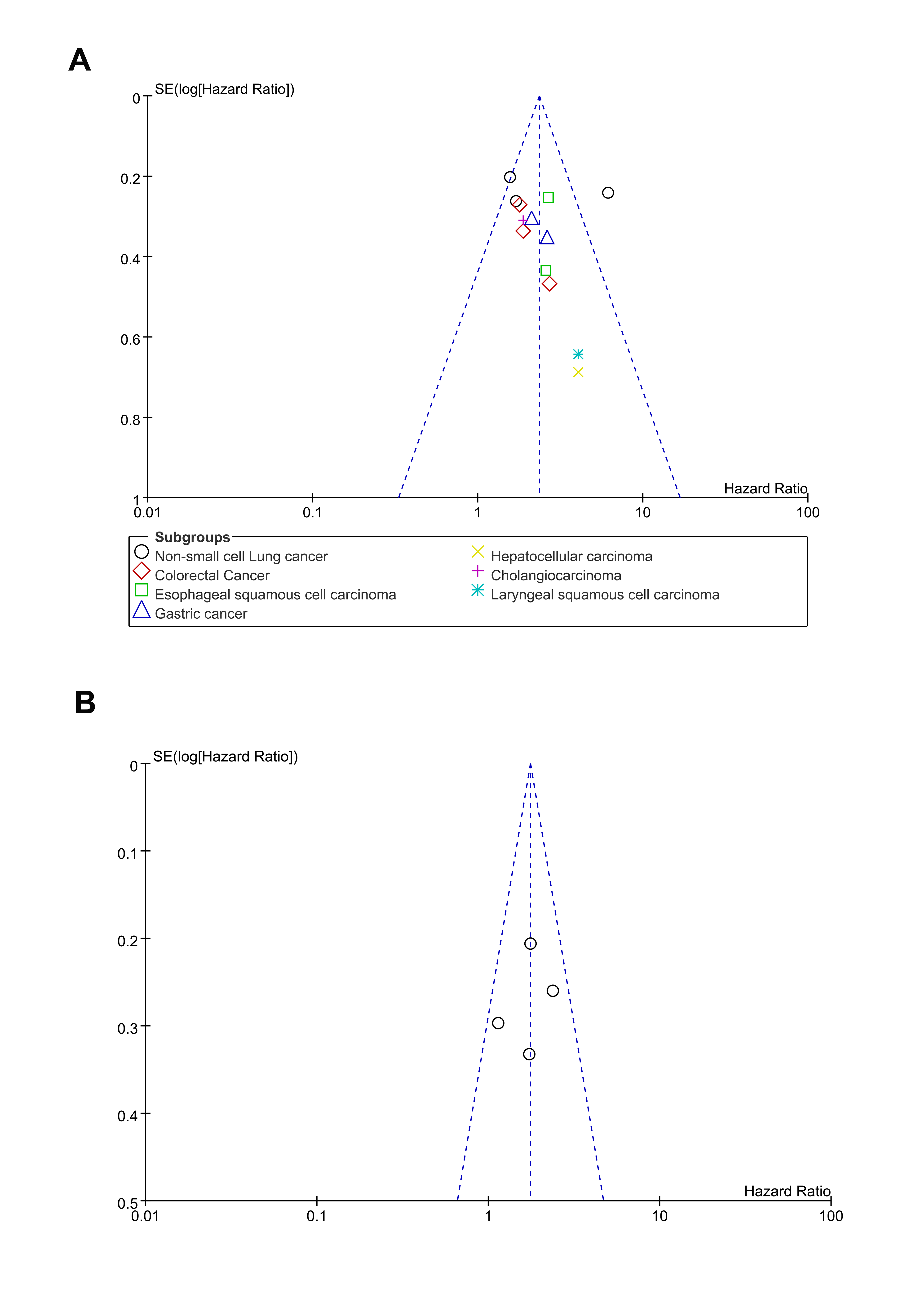

Supplement: Supplementary file 3 — Fig S3 [file JCMM-24-9507-s003.tif]
