# Supplementary material for: Diagnostic and prognostic value of circular RNA CDR1as/ciRS‐7 for solid tumours: A systematic review and meta‐analysis
Source: J Cell Mol Med. 2020 Aug 12;24(17):9507–17. doi: 10.1111/jcmm.15619 (PMC7520288; doi:10.1111/jcmm.15619)
Supplement: Supplementary file 4 — Table S1‐S5 [file JCMM-24-9507-s004.docx]

**Supplemental Table 1. Main baseline characteristics and outcomes of the included studies for diagnosis analysis**

Abbreviations: *Sen*, sensitivity; *Spe*, specificity; AUC, Area Under Curve; HCC, Hepatocellular carcinoma; CHOL, Cholangiocarcinoma; CC, Colorectal cancer; ESCC, Esophageal squamous cell carcinoma; TNBC, Triple-negative breast cancer; OS, Osteosarcoma; NSCLC, Non-small cell lung cancer; LSCC, Laryngeal squamous cell carcinoma; CESC, Cervical squamous cell carcinoma; (a) Specimen from tissue ; (b) Specimen from plasma

| Study | Year | Cancer type | Specimen | Method | Sample size | | *Sen* | *Spe* | AUC |
| --- | --- | --- | --- | --- | --- | --- | --- | --- | --- |
|  |  |  |  |  | Case | Control |  |  |  |
| Yu | 2016 | HCC | Tissue | qRT-PCR | 35 | 35 | 0.71 | 0.69 | 0.73 |
| Jiang | 2017 | CHOL | Tissue | qRT-PCR | 54 | 54 | 0.76 | 0.87 | 0.86 |
| Tang | 2017 | CC | Tissue | qRT-PCR | 40 | 40 | 0.73 | 0.73 | 0.72 |
| Weng | 2017 | CC | Tissue | qRT-PCR | 40 | 40 | 0.50 | 0.68 | 0.63 |
| Xu2 | 2017 | HCC | Tissue | qRT-PCR | 46 | 46 | 0.54 | 0.72 | 0.68 |
| Li | 2018 | ESCC | Tissue | qRT-PCR | 123 | 123 | 0.63 | 0.66 | 0.67 |
| Meng | 2018 | ESCC | Tissue | qRT-PCR | 60 | 60 | 0.68 | 0.83 | 0.70 |
| Sang1 | 2018 | ESCC | Tissue | qRT-PCR | 86 | 86 | 0.59 | 0.81 | 0.65 |
| Sang2 | 2018 | TNBC | Tissue | qRT-PCR | 32 | 32 | 0.94 | 1.00 | 0.99 |
| Xu1 | 2018 | OS | Tissue | qRT-PCR | 38 | 18 | 0.82 | 0.83 | 0.86 |
| Yan | 2018 | NSCLC | Tissue | qRT-PCR | 132 | 132 | 0.68 | 0.80 | 0.83 |
| Zhang1 | 2018 | LSCC | Tissue | qRT-PCR | 30 | 30 | 0.90 | 0.97 | 0.98 |
| Zhang2 | 2018 | NSCLC | Tissue | qRT-PCR | 60 | 20 | 0.68 | 0.95 | - |
| Cheng | 2019 | CESC | Tissue | qRT-PCR | 40 | 40 | 0.80 | 0.70 | 0.80 |
| Fan(a) | 2019 | ESCC | Tissue | qRT-PCR | 65 | 50 | 0.54 | 0.64 | 0.62 |
| Fan(b) | 2019 | ESCC | Plasma | qRT-PCR | 50 | 50 | 0.92 | 0.80 | 0.89 |

**Supplemental Table 2. Main baseline characteristics and outcomes of the included studies for prognosis analysis**

Abbreviations: CHOL, Cholangiocarcinoma; NSCLC, Non-small cell lung cancer; CC, Colorectal cancer; HCC, Hepatocellular carcinoma；ESCC, Esophageal squamous cell carcinoma; GC, Gastric cancer; LSCC, Laryngeal squamous cell carcinoma; (a) training cohort; (b) validation cohort

| Study | Year | Cancer type | Specimen | Method | CDR1as expression | | Follow up |
| --- | --- | --- | --- | --- | --- | --- | --- |
|  |  |  |  |  | High | Low | (month) |
| Jiang | 2017 | CHOL | Tissue | qRT-PCR | 24 | 30 | 41 |
| Su | 2017 | NSCLC | Tissue | qRT-PCR | 77 | 51 | 60 |
| Tang | 2017 | CC | Tissue | qRT-PCR | 91 | 91 | 60 |
| Weng(a) | 2017 | CC | Tissue | qRT-PCR | 76 | 77 | 83 |
| Weng(b) | 2017 | CC | Tissue | qRT-PCR | 89 | 76 | 117 |
| Xu2 | 2017 | HCC | Tissue | qRT-PCR | 48 | 47 | 63 |
| Li | 2018 | ESCC | Tissue | qRT-PCR | 61 | 62 | 92 |
| Pan(a) | 2018 | GC | Tissue | qRT-PCR | 50 | 52 | 60 |
| Pan(b) | 2018 | GC | Tissue | qRT-PCR | 83 | 71 | 58 |
| Yan | 2018 | NSCLC | Tissue | qRT-PCR | 66 | 66 | 85 |
| Zhang1 | 2018 | LSCC | Tissue | qRT-PCR | 15 | 15 | 60 |
| Zhang2 | 2018 | NSCLC | Tissue | qRT-PCR | 41 | 19 | 82 |
| Fan | 2019 | ESCC | Tissue | qRT-PCR | 25 | 25 | 32 |

**Supplemental Table 3. The regulatory mechanisms of CDR1as/ciRS-7 in solid tumors.**

Abbreviations: HCC, Hepatocellular carcinoma; CHOL, Cholangiocarcinoma; CC, Colorectal cancer; ESCC, Esophageal squamous cell carcinoma; TNBC, Triple-negative breast cancer; OS, Osteosarcoma; NSCLC, Non-small cell lung cancer; GC, Gastric cancer; LSCC, Laryngeal squamous cell carcinoma; CESC, Cervical squamous cell carcinoma.

| Study | Year | Cancer type | Expression | Regulatory mechanism |
| --- | --- | --- | --- | --- |
| Yu | 2016 | HCC | Upregulated | miR-7-CCNE1/PIK3CD |
| Jiang | 2017 | CHOL | Upregulated | / |
| Su | 2017 | NSCLC | Upregulated | miR-7-NF-κB signaling |
| Tang | 2017 | CC | Upregulated | miR-7-EGFR/IGF1R |
| Weng | 2017 | CC | Upregulated | miR-7-EGFR/RAF1 |
| Xu2 | 2017 | HCC | Upregulated | miR-7-PI3K-mTOR |
| Li | 2018 | ESCC | Upregulated | miR-7-HOXB13- NF-κB/p65 |
| Meng | 2018 | ESCC | Upregulated | / |
| Pan | 2018 | GC | Upregulated | miR-7-PTEN/PI3K/AKT signaling |
| Sang1 | 2018 | ESCC | Upregulated | miR-876-5p-MAGE-A family |
| Sang2 | 2018 | TNBC | Upregulated | miR-1299-MMP2/17 |
| Xu1 | 2018 | OS | Upregulated | miR-7-EGFR/CCNE1/PIK3CD/RAF1 |
| Yan | 2018 | NSCLC | Upregulated | / |
| Zhang1 | 2018 | LSCC | Upregulated | miR-7-CCNE1/PIK3CD |
| Zhang2 | 2018 | NSCLC | Upregulated | miR-7-EGFR/CCNE1/PIK3CD |
| Cheng | 2019 | CESC | Upregulated | miR-7-PTEN/PI3K/AKT signaling |
| Fan | 2019 | ESCC | Upregulated | miR-7-IPO11/SNCA/CALU/CRY2 |

**Supplemental Table 4. Methodological quality of included studies for prognosis based on Newcastle-Ottawa Scale (NOS)**

Scoring items: ①Representativeness of the exposed cohort；②Selection of the non-exposed cohort; ③Ascertainment of exposure; ④Outcome was not present at start of study; ⑤Comparability of cohorts on the basis of the design or analysis; ⑥Assessment of outcome; ⑦Was follow-up long enough for outcomes to occur; ⑧Adequacy of follow up

| Study | ① | ② | ③ | ④ | ⑤ | ⑥ | ⑦ | ⑧ | Score |
| --- | --- | --- | --- | --- | --- | --- | --- | --- | --- |
| Jiang 2017 | 1 | 1 | 1 | 0 | 1 | 1 | 1 | 0 | 6 |
| Su 2017 | 1 | 1 | 1 | 0 | 1 | 1 | 1 | 0 | 6 |
| Tang 2017 | 1 | 1 | 1 | 0 | 0 | 1 | 1 | 0 | 5 |
| Weng(a) 2017 | 1 | 1 | 1 | 0 | 1 | 1 | 1 | 1 | 7 |
| Weng(b) 2017 | 1 | 1 | 1 | 0 | 1 | 1 | 1 | 1 | 7 |
| Xu2 2017 | 1 | 1 | 1 | 0 | 1 | 1 | 1 | 1 | 7 |
| Li 2018 | 1 | 1 | 1 | 0 | 1 | 1 | 1 | 1 | 7 |
| Pan(a) 2018 | 1 | 1 | 1 | 0 | 0 | 1 | 1 | 0 | 5 |
| Pan(b) 2018 | 1 | 1 | 1 | 0 | 0 | 1 | 1 | 0 | 5 |
| Yan 2018 | 1 | 1 | 1 | 0 | 1 | 1 | 1 | 1 | 7 |
| Zhang1 2018 | 1 | 1 | 1 | 0 | 1 | 1 | 1 | 0 | 6 |
| Zhang2 2018 | 1 | 1 | 1 | 0 | 1 | 1 | 1 | 0 | 6 |
| Fan 2019 | 1 | 1 | 1 | 0 | 1 | 1 | 1 | 0 | 6 |

**Supplemental Table 5. Website address of the retrieval databases.**

| **Database** | **Website address** |
| --- | --- |
| PubMed | <https://pubmed.ncbi.nlm.nih.gov/> |
| Embase | <https://www.embase.com/> |
| the Cochrane library | <https://www.cochranelibrary.com/> |
| Web of Science | <https://webofscience.com/> |
